# Atypical clinical presentation associated with Castleman disease: a case report and review of the literature

**DOI:** 10.3389/fmed.2025.1626722

**Published:** 2025-08-21

**Authors:** Weiwei Hu, Yuke Kong, Bianxiang Hu, Jun Ai, Peng Zhang

**Affiliations:** ^1^Department of Nephrology, The Second Hospital & Clinical Medical School, Lanzhou University, Lanzhou, China; ^2^Department of Nephrology, Nanfang Hospital, Southern Medical University, Guangzhou, China; ^3^Department of Pathology, The Second Hospital & Clinical Medical School, Lanzhou University, Lanzhou, China

**Keywords:** Castleman disease (CD), idiopathic multicentric Castleman disease (iMCD), atypical clinical presentation, acute kidney injury (AKI), lymph node

## Abstract

**Background:**

Castleman disease (CD) is a group of rare and complicated diseases characterized by systemic inflammation, lymphadenopathy, and multiorgan involvement. It is often misdiagnosed as an infection, an autoimmune disease, or a malignant cancer.

**Case presentation:**

In this case, we report a 33-year-old Chinese male patient who was diagnosed with idiopathic multicentric Castleman disease (iMCD). The patient initially presented with intermittent fever and abdominal pain, followed by the development of abdominal and thoracic cavity effusions, anemia, thrombocytopenia, increased D-dimer levels, and acute kidney injury (AKI). Notably, there was an absence of significant lymphadenopathy in the early stages of disease progression. The final diagnosis was idiopathic multicentric Castleman disease (iMCD).

**Conclusion:**

This case emphasizes the importance of considering iMCD in patients with persistent fever and systemic inflammation, even in the absence of noticeable lymphadenopathy. We should monitor the progression of the disease and adopt a developmental perspective. Different stages of the disease can have different clinical manifestations. Diagnosis requires a comprehensive assessment of clinical, laboratory, and histopathological findings. The rarity and complexity of iMCD can pose diagnostic challenges, especially for healthcare providers who are not familiar with the disease, potentially leading to misdiagnosis. To prevent misdiagnosis, healthcare providers must maintain a high index of suspicion for CD in patients with prolonged fever and no significant lymph node enlargement. Timely recognition and accurate diagnosis are critical for initiating appropriate treatments and improving patient outcomes. Enhancing awareness of iMCD among healthcare providers is essential for early detection and effective management.

## Background

Castleman disease (CD) is a clinically extraordinary disorder that is always caused by abnormal proliferation of lymphoid tissues. Benjamin Castleman identified unicentric Castleman disease (UCD) in 1954. Multicentric Castleman disease (MCD) was first described in 1985 and is a potentially life-threatening systemic disease (1). The pathogenesis of CD is unknown and probably related to viral infections, such as human immunodeficiency virus, human herpesvirus 8 (HHV-8), and interleukin 6 (IL-6) overexpression. Meanwhile, according to the anatomic distribution, CD is classified into two subgroups: unicentric Castleman disease (UCD) and multicentric Castleman disease (MCD) (2). Patients with MCD who test negative for human herpesvirus type 8 (HHV-8) are diagnosed with idiopathic MCD (iMCD). In contrast, UCD often presents as limited lymph node enlargement, preferably in the mediastinum and neck, and usually prompts consultation due to compression symptoms or occasional enlarged lymph nodes (3). In addition, patients with iMCD often present with multiple lymph node enlargements, usually involving various regions. They may also experience clinical symptoms such as fatigue, intermittent fever, weight loss, anemia, and hepatosplenomegaly. Furthermore, some MCD cases can lead to organ failure and even death (3).

In this case report, we describe the clinical case of a young man aged 33 years with an atypical clinical presentation occurring at different stages. The patient initially presented with intermittent fever and abdominal pain, followed by abdominal and thoracic cavity effusions, anemia, thrombocytopenia, increased D-dimer levels, and acute kidney injury (AKI). Notably, he lacked significant lymphadenopathy during his first hospitalization, which led to a misdiagnosis.

This case highlights the importance of considering Castleman disease as a potential diagnosis in patients with unexplained systemic symptoms or lymphadenopathy, particularly when common causes have been excluded. It emphasizes the need for healthcare providers to maintain a high index of suspicion for Castleman disease, especially if the presentation is atypical or if there is no significant lymphadenopathy. Early diagnosis and timely initiation of appropriate treatment are essential to achieve positive outcomes and prevent unnecessary morbidity and mortality associated with Castleman disease.

## Case presentation

This case report describes a 33-year-old male patient with no significant medical history. In the early stages of the disease, the patient was admitted to the gastroenterology department of a local hospital with intermittent low-grade fever, nausea, and abdominal pain lasting approximately 2 weeks, without any lymph node enlargement. The patient’s abdominal pain was localized to the upper stomach area, and the pain was not affected by changes in position. Murphy’s sign was negative during the inpatient medical examinations. After the examinations, he was diagnosed with abdominal and pleural effusions, anemia, thrombocytopenia, increased D-dimer levels, and acute kidney injury (AKI). The patient’s AKI was characterized by a progressive increase in blood creatinine levels with oliguria. As a result, the patient was referred to the nephrology department of the local hospital for dialysis. The patient received symptomatic supportive treatments with antispasmodics and analgesics, anti-infective therapy, thoracic and abdominal puncture drainage, diuretic therapy, two platelet transfusions, and two hemodialysis treatments at the local hospital. The patient’s indicators improved for a short time but then deteriorated, and the outcome seemed to be unsatisfactory. To clarify the etiology of the multisystem damage and to receive more effective treatment for further diagnosis and management, the patient was referred to the nephrology department of Nanfang Hospital in June 2024.

On admission to our hospital, the patient’s heart rate was increased to 112 beats per minute, and his temperature was 37.5 °C, while his blood pressure and oxygen levels were normal. In addition, respiratory sounds were decreased bilaterally, the abdomen was distended with fluid accumulation, and lymph nodes were palpable in the right inguinal and left mandible regions. After a detailed consultation, we confirmed that the patient had no history of recent travel, medication administration, or changes in his home living environment. Moreover, in the course of receiving treatment at the local hospital, he developed new-onset thrombocytopenia and AKI. The AKI (pre-renal or intrinsic) was characterized by a progressive increase in blood creatinine levels, from an initial level of 144.7umol/L to 727umol/L with oliguria. Meanwhile, after admission to our hospital, the patient’s laboratory tests showed mild anemia (hemoglobin level, 96 g/L; normal range 120–150 g/L), slightly low platelet count (PLT 85 × 10^9^/L; normal range 150–300 × 10^9^/L), hypoalbuminemia (serum albumin level, 33.1 g/L; normal range 40–55 g/L), and renal failure (serum creatinine level, 137umol/L; normal range 57–97umol/L; urea nitrogen level 8.6 mmol/L; normal range 3.1–8.0 mmol/L), as well as elevated inflammatory parameters (white cell count 16.9 × 10^9^/L; normal range 4.0–10.0 × 10^9^/L; Creactive protein (CRP) 140.84 mg/L; normal range < 10; and PCT 3.930 ng/mL; normal range < 0.05). In addition, D-dimer was elevated (14.66 μg/mL), and significant proteinuria was noted (urinary protein level, 7.59 g/24 h).

There were no biological signs of hemolysis, as indicated by normal lactate dehydrogenase levels and elevated haptoglobin levels. Meanwhile, the immunological indicators—including antinuclear antibody, rheumatoid factor, antineutrophil cytoplasmic antibody, and anti-glomerular basement membrane antibody—were normal. In addition, the blood tests for infectious indicators, such as serological testing for CMV cytomegalovirus, Epstein–Barr virus (EBV), toxoplasma, HHV-8, hepatitis B virus, hepatitis C virus, and human immunodeficiency virus (HIV), were all negative. Furthermore, the patient’s serum immunofixation electrophoresis, thyroid function, tuberculosis tests, and tumor-related markers, as well as ADA value in ascites, were all negative. In addition, there was no evidence suggestive of atypical hemolytic-uremic syndrome. Bone marrow aspiration did not reveal any certain cause for the thrombocytopenia.

The ultrasound report indicated multiple enlarged lymph nodes bilaterally in the neck, axilla, groin, and para-abdominal aorta regions.

The abdominal CT scan revealed multiple enlarged lymph nodes in the bilateral shoulders and neck, bilateral axilla, mediastinum, bilateral lung hilar, abdominal cavity, retroperitoneum, and bilateral inguinal regions. It also showed fluid accumulation in both thoracic cavities and an enlarged spleen.

Positron emission tomography (PET) showed multiple lymph nodes with moderate hypermetabolism at the lateral side of the neck, axillary, and right lung apex, as well as a hypermetabolic spleen.

**Figure 1:**
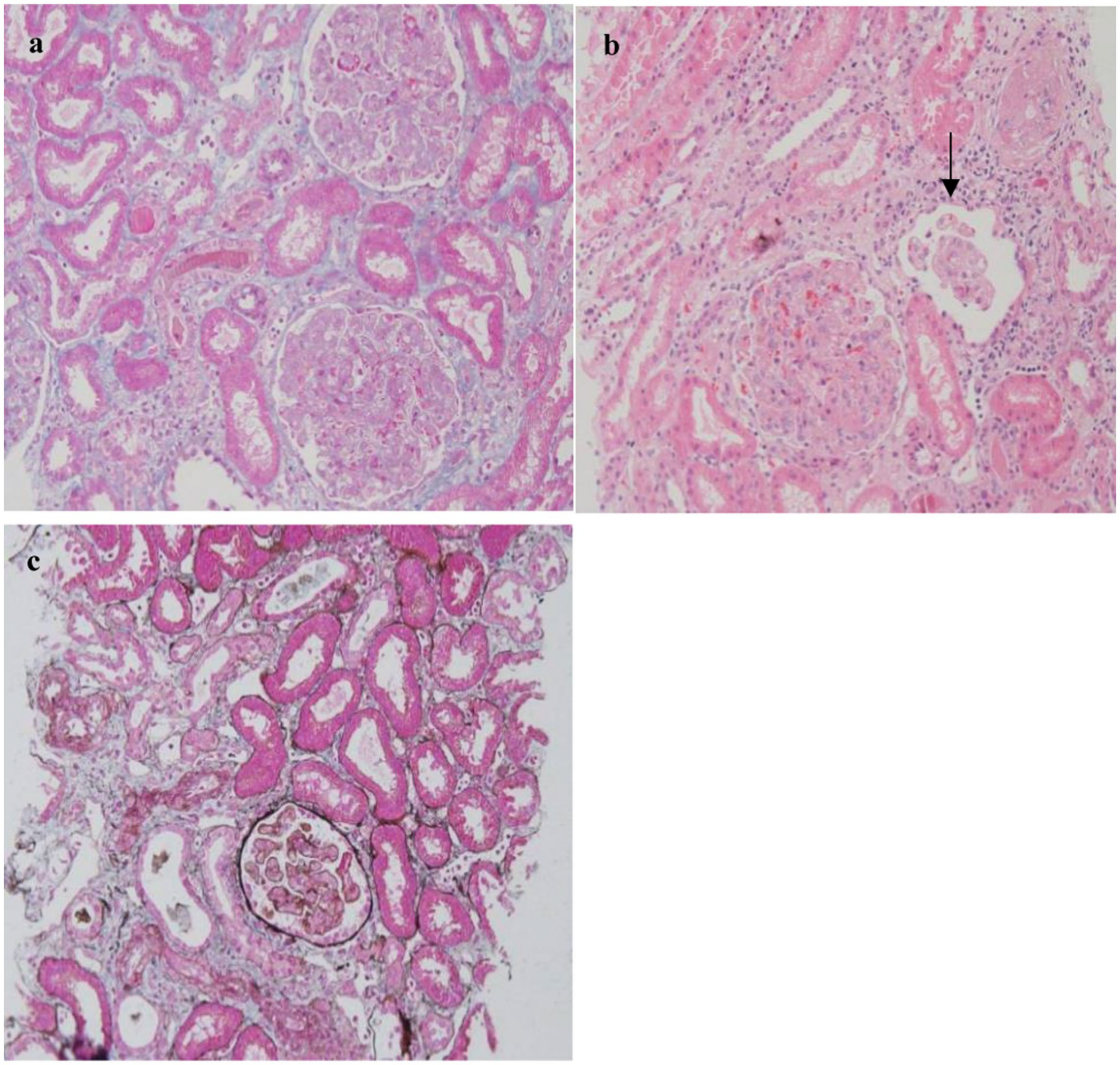
Histopathological findings in renal biopsies from the patient with MCD renal **(a–c)**. **(a)** Diffuse endocapillary proliferation and endothelial swelling (hematoxylin and eosin stain [H&E, ×200]). **(b)** Partial glomerular atrophy. **(c)** Glomerular atrophy, thickening of the tegmental area of the glomerulus (Hexa-ammonia silver stain, ×200).

Finally, when the patient was in relatively stable condition, a renal biopsy was performed. The pathology of the biopsy showed TMAlike lesions (Figure 1). Light microscopy of the renal biopsy showed a partial increase in glomerular volume, with diffuse moderate to segmental severe widening of the tethered zone, accompanied by cellular and stromal hyperplasia. Small amounts of red blood cell retention and thrombosis were also observed. Focal renal tubular atrophy with a protein tubular pattern was observed. Meanwhile, light microscopy showed diffuse endothelial swelling, no thrombus in the capillary loops, no fracture of the glomerular basement membrane (GBM), no significant thickening, no nail changes, and predominantly pseudo-double tracking sign formation and double contouring. There were no crescents, fibrinoid necrosis, or overt fibrosis. At the same time, electron microscopy of the renal biopsy showed findings consistent with changes in thrombotic microangiopathy. Immunofluorescence (IF) was negative for IgG, IgM, IgA, C3, C1q, kappa, and lambda. Furthermore, along individual capillary collaterals and small renal arterioles, C4D was positively expressed. A renal biopsy was performed, which showed TMAlike lesions.

The patient was admitted with intermittent fever, swelling, abdominal pain, and diarrhea this time, so he was treated with antibiotics, gastrointestinal support, diuretics, and other symptomatic treatments. Despite the above treatments, the patient’s condition did not improve, and the exact cause of the infection remained unclear. In addition, the patient was found to have multiple enlarged lymph nodes on examination during this admission. Therefore, to find out the cause of the disease, a biopsy of the cervical lymph nodes was performed.

**Figure 2:**
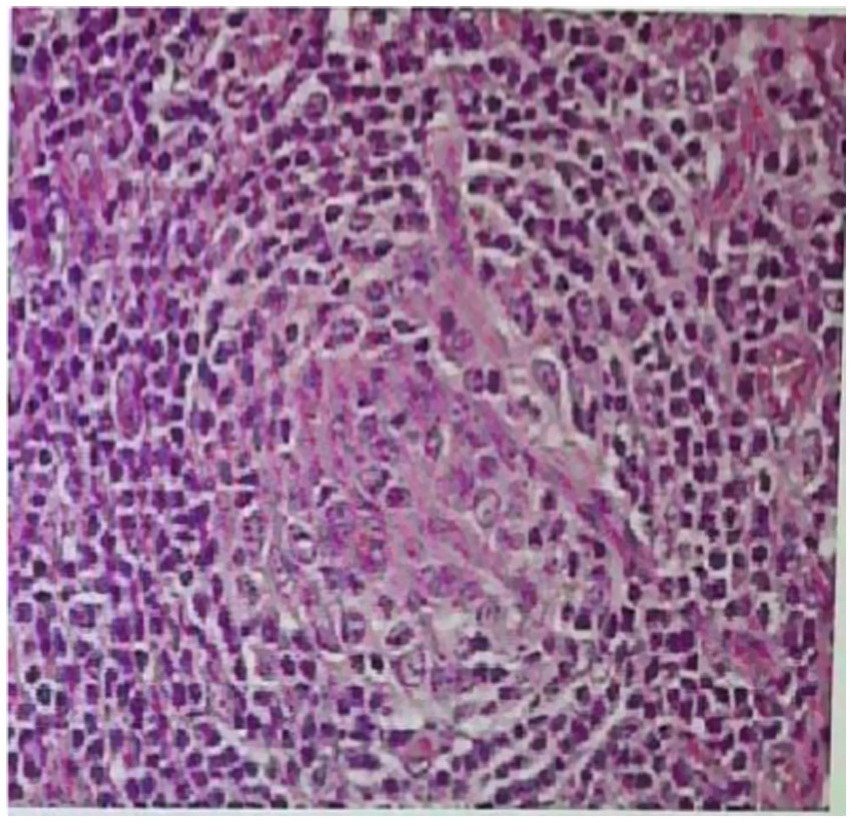
A lymph node biopsy showed evidence of iMCD-TAFRO with plasma cell infiltration as the main pathological manifestation.

Following comprehensive clinical examinations, the patient underwent a lymph node biopsy, which was suggestive of Castleman disease, plasma cell type (shown in Figure 2). The histological features of the patient’s left cervical lymph node were as follows: the structure of the presented tissue showed scattered lymphoid follicles with atrophied germinal centers surrounded by small lymphocytes arranged in an onion-skin pattern, as well as scattered plasma cells in the interfollicular area. Fortunately, the pathology results from the left cervical lymph node biopsy provided a definitive diagnosis: the patient was diagnosed with CD. After a series of examinations, the patient was ultimately diagnosed with iMCD-TAFRO, AKI, upper respiratory tract infection, abdominal effusion, pleural effusions, and enlarged spleen (4). Due to the diagnosis of CD, the patient was transferred to the hematology department for further specialized treatment. In the subsequent telephone follow-up, we received information that the patient had received stuximab in combination with thalidomide, cyclophosphamide, and dexamethasone as the main therapeutic regimen in the hematology department of the local hospital. At the subsequent out-of-hospital follow-up in January 2025, the patient’s renal function, albumin levels, and urinary proteins had fully returned to normal, and we were extremely gratified by these positive results. We will continue to monitor this case closely.

## Discussion

CD is an uncommon lymphoproliferative disorder characterized by focal or generalized lymphadenopathy (3). In 2017, the Castleman Disease Collaborative Network (CDCN) proposed a unified system for classifying CD. CD is classified into two subgroups according to the involvement of lymph nodes: unicentric Castleman disease (UCD) and multicentric Castleman disease (MCD). MCD is further divided into two types based on human herpesvirus 8 (HHV-8) infection: HHV-8-positive MCD and HHV-8-negative (idiopathic) MCD (iMCD). In addition, the concept of asymptomatic MCD (aMCD) has been reported. This variant shows histological features similar to Castleman disease but lacks inflammatory findings and does not meet the diagnostic criteria established by the CDCN (5).

The diagnosis of iMCD requires meeting both of the following major criteria: (1) Presence of ≥2 groups of enlarged lymph nodes (>1 cm in short-axis diameter) confirmed by imaging (CT/PET-CT) or pathological examination; (2) Rule out other similar diseases, including infections (e.g., HIV and HHV-8), malignant tumors (e.g., lymphoma and metastatic carcinoma), and autoimmune diseases (e.g., lupus and rheumatoid arthritis). In addition to the two major criteria, the diagnosis of iMCD requires at least 2 out of 11 minor criteria, with at least 1 being a laboratory criterion. The laboratory criteria were as follows: (1) Elevated CRP (>10 mg/L) or ESR (>15 mm/h); (2) anemia (hemoglobin < 12.5 g/dL (male individuals), <11.5 g/dL (female individuals)); (3) thrombocytopenia (platelet < 150 k/μL) or thrombocytosis (platelet count > 400 k/μL); (4) hypoalbuminemia (albumin < 3.5 g/dL); (5) renal dysfunction (eGFR < 60 mL/min/1.73 m2); and (6) polyclonal hypergammaglobulinemia (total gamma globulin or immunoglobulin G > 1700 mg/dL). The clinical criteria were as follows: (1) Constitutional symptoms: night sweats, fever (>38 °C), weight loss, or fatigue; (2) large spleen and/or liver; (3) fluid accumulation: edema, anasarca, ascites, or pleural effusion; (4) eruptive cherry hemangiomatosis or violaceous papules; and (5) lymphocytic interstitial pneumonitis (6). Diagnosis of iMCD is often difficult, with a high degree of symptomatic heterogeneity. iMCD can be classified into the plasma cell type, the hematocrit type, and the mixed type according to the pathological features of the lymph nodes. Meanwhile, it can be further categorized based on clinical phenotypes into iMCD-not otherwise specified (iMCD-NOS) and iMCD-TAFRO (7, 8). From a therapeutic point of view, an accurate diagnosis of the different types of CD is very important, as it facilitates the subsequent development of a therapeutic regimen. Our case was ultimately diagnosed as iMCD-TAFRO. TAFRO syndrome is a systemic inflammatory disease characterized by thrombocytopenia (T), generalized anasarca (A), fever (F), reticulin fibrosis (R), and organ enlargement (O) and is considered to be a specific subtype of HHV-8-negative iMCD. Renal impairment is a common clinical symptom of TAFRO syndrome. When diagnosing iMCD, it is important to distinguish it from acute EBV infection, HIV infection, tuberculosis, systemic lupus erythematosus, rheumatoid arthritis, thrombocytopenic purpura, lymphoma, and POEMS syndrome. Furthermore, we have also summarized a table listing some of the diseases in the differential diagnosis of Castleman disease (CD) in terms of anatomical location, vascular and lymphatic symptoms, clinical symptoms, organomegaly, and systemic dysfunction to provide a comparative overview (Table 1).

**Table 1:** The differential diagnosis of Castleman disease (CD).

Item	UCD	HHV8-MCD	POEMS-MCD	iMCD-TAFRO
Anatomical location	Intra- or extra-thoracic	Multiple lymph nodes and tissues	Multiple lymph nodes and tissues	Multiple lymph nodes and tissues
Vascular and lymphatic symptoms	Central lymphadenopathy is the most common and it increases in size	Multiple lymphadenopathy; pancytopenia	Multiple lymphadenopathy; pancytopenia	Multiple lymphadenopathy; pancytopenia
Clinical symptoms	Symptoms associated with localized anatomical compression are prevalent	Fever, night sweats, generalized swelling, weight loss	Fever, night sweats, generalized swelling, weight loss	Fever, night sweats, generalized swelling, weight loss
Organomegaly	Rarely	None or rare	Hepatomegaly, splenomegaly	Hepatomegaly, splenomegaly
Systemic dysfunction	None or rare	Abnormal kidney and liver function	Polyneuropathy, endocrine diseases, skin changes	Abnormal kidney and liver function

UCD, unicentric Castleman disease; MCD, multicentric Castleman disease.

CD has been estimated to occur at approximately 25 cases per million person-years, corresponding to fewer than 5,200 cases per year in the USA (2). There are only a few epidemiological studies on CD. MCD occurs slightly more often in male individuals than in female individuals, whereas UCD shows no significant sex difference. The average age at diagnosis for UCD is usually younger (40 years) compared to MCD (60 years) (4, 5). According to a recent study, the incidence and prevalence of iMCD in the USA are 3.4 and 6.9 cases per million per year, while the incidence of iMCD in Japan is 2.4 cases per million (1). The evaluated incidence of CD in the United States is between 4,300 and 5,200 cases annually. A study of 145 HIV-negative patients with Castleman disease in Beijing, China, clinically classified 69 patients (47.6%) as having UCD and 76 patients (52.4%) as having MCD (3). The complex symptomatology is mostly because of the overproduction of interleukin-6 or the dysregulation of IL-6-related signaling pathways (9).

The pathogenesis of CD is unknown. Viral infections are regarded as significant factors in the pathogenesis of CD, and some researchers have already pointed out that there is a close relationship between HHV-8 and MCD (3). Meanwhile, in Talat’s study, HHV-8 was detected in 46 of 49 (93.9%) patients with MCD, compared to 13 patients with UCD (33.3%). In our case, the HIV test result was negative (4). Regarding histopathological examination, UCD most commonly presents as the hyaline vascular variant (90%), whereas in MCD, the plasmacytoid variant is more commonly observed (4).

The typical pathological features of CD include mantle zone lymphocytes arranged in concentric rings around the follicles, giving them a target-like morphology (5). The systemic features and widespread lymphadenopathy of MCD may mimic various conditions, including autoimmune disorders, acute infectious diseases, POEMS syndrome, and malignancies, particularly lymphoma. The presence of positive diagnostic criteria, histopathological lymph nodes, and enlarged lymph nodes aids in the diagnosis (6). When we diagnose CD, pathology is the gold standard, and we also need to rule out HIV-associated lymphadenopathy, autoimmune diseases, lymphomas, and other diseases.

Pertusa Mataix, R.’s study had pointed out that histopathological examination through biopsy remains crucial for diagnosis, as it helps in identifying characteristic features of the disease. Blood tests, including markers of inflammation and specific cytokine levels (IL-6, IL-10, CRP, VEGF, and beta-2 microglobulin), are also conducted to evaluate systemic involvement (1). Performing these tests helps in distinguishing Castleman disease from other similar conditions and tailoring appropriate treatment strategies (9). Meanwhile, Koa’s study emphasizes the importance of 18F-fluorodeoxyglucose positron emission tomography (18F-FDG PET)/CT scans in diagnosing Castleman disease. He believes that this examination provides detailed metabolic insights, revealing glucose uptake patterns that help distinguish Castleman disease from other lymphoproliferative disorders (10). It is crucial for assessing disease extent, staging, and monitoring treatment responses by tracking changes in metabolic activity (11).

Up to 50% of patients with CD may develop renal complications. The most common finding on a kidney biopsy is TMA, followed by amyloid A amyloidosis (7). The remaining findings are less common and include membranoproliferative glomerulonephritis, IgA nephropathy, focal segmental glomerulosclerosis, and membranous glomerulopathy (5, 8). A study showed that the link between renal TMA and Castleman disease is related to increased serum VEGF levels and decreased VEGF expression in the glomeruli (12).

CD can present with a variety of findings, resulting in delayed or incorrect diagnosis. Diagnosis is challenging due to its broad differential diagnosis, clinical heterogeneity, and limited pathophysiologic understanding (11). The systemic presentation and multiple sites of lymphadenopathy in MCD must be distinguished from a variety of autoimmune, acute infectious, and malignant conditions (13). In Mathew’s article, it is pointed out that because of the asymptomatic symptoms, it is difficult to distinguish UCD from other diseases with swollen lymph nodes, potentially leading to misdiagnosis or delayed identification (9, 14).

Although hematology specialists have a certain understanding of CD, there remains a lack of awareness among other doctors in different departments, especially when admitting a patient with multiple lymphomas.

From the available literature, it is clear that the best choice of treatment for UCD is surgical resection, if it is possible. With complete surgical resection, almost all symptoms can be resolved and laboratory abnormalities can return to normal (15). Consensus guidelines for treating UCD include siltuximab, tocilizumab, rituximab, corticosteroids, and other therapies (13).

Finally, the strength of this study lies in the fact that, despite the patient’s varied clinical presentation at different stages of the disease and the presence of a number of comorbidities, an accurate diagnosis was ultimately made. The information presented helped guide appropriate diagnosis and treatment, resulting in a favorable outcome. Certainly, our case report has some limitations. Firstly, we were unable to access the detailed results of the examinations performed during the patient’s initial hospitalization at the local hospital, having only the critical and conclusive information from the discharge summary. This limitation may have complicated the differential diagnosis. Secondly, we did not perform a lymph node biopsy in time after the patient was admitted to our hospital, which led to a number of detours in the treatment process. Lastly, after the patient’s diagnosis was confirmed, treatment was administered at the other hospital. Although we concluded that the therapeutic effect was satisfactory, we did not have a clear idea about the specific drug dosages or the changes in the indicators before and after treatment.

This case demonstrates the importance of monitoring disease progression and adopting a developmental view, as a single disease can present with different clinical features at various stages. In other words, the diagnosis requires a comprehensive assessment of clinical, laboratory, and histopathological findings, as well as consideration of the overall stage of disease development. The rarity and complexity of iMCD make it challenging to diagnose, especially for healthcare providers unfamiliar with the condition, and may lead to misdiagnosis. To avoid this, healthcare providers must maintain a high index of suspicion for iMCD in patients with prolonged fever and no significant lymphadenopathy. Thorough investigations are essential in such suspicious cases. To initiate appropriate treatment and improve patient outcomes, timely recognition and accurate diagnosis are critical. For early recognition and effective management, increased awareness of iMCD among healthcare providers is essential.

## Data Availability

The original contributions presented in the study are included in the article/supplementary material, further inquiries can be directed to the corresponding author.
